# Supplementary material for: Heavy Metals in Unprocessed or Minimally Processed Foods Consumed by Humans Worldwide: A Scoping Review
Source: Int J Environ Res Public Health. 2022 Jul 16;19(14):8651. doi: 10.3390/ijerph19148651 (PMC9319294; doi:10.3390/ijerph19148651)
Supplement: Supplementary file 1 [file ijerph-19-08651-s001.zip › Figure S1_Map 5 to 10_HMS concentration in the least reported food groups.pdf]

Figure S1. Heavy metals concentration in parts per million (ppm) in the least reported food groups worldwide

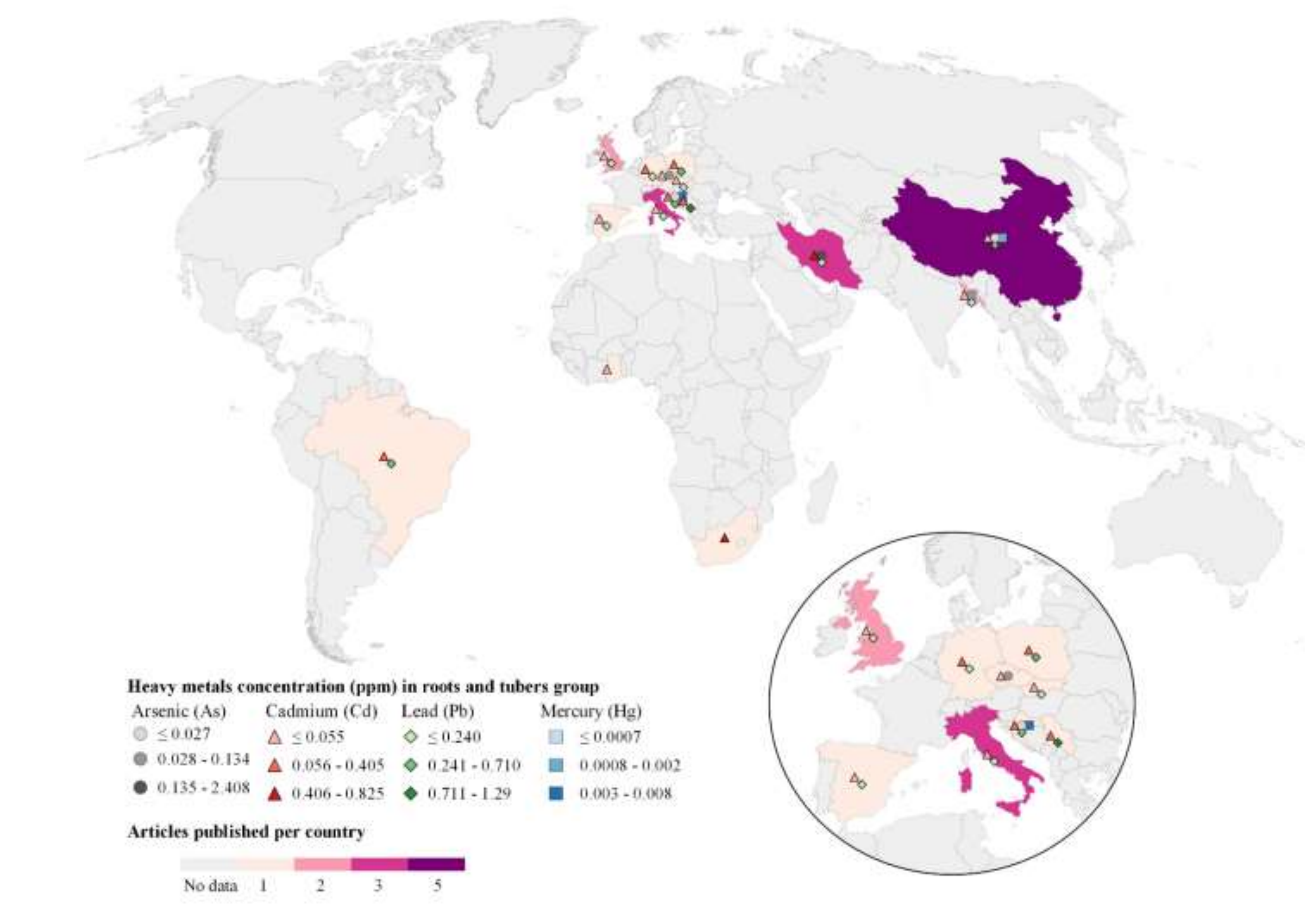

Map 5. Heavy metals concentration in Roots and tubers food group by country

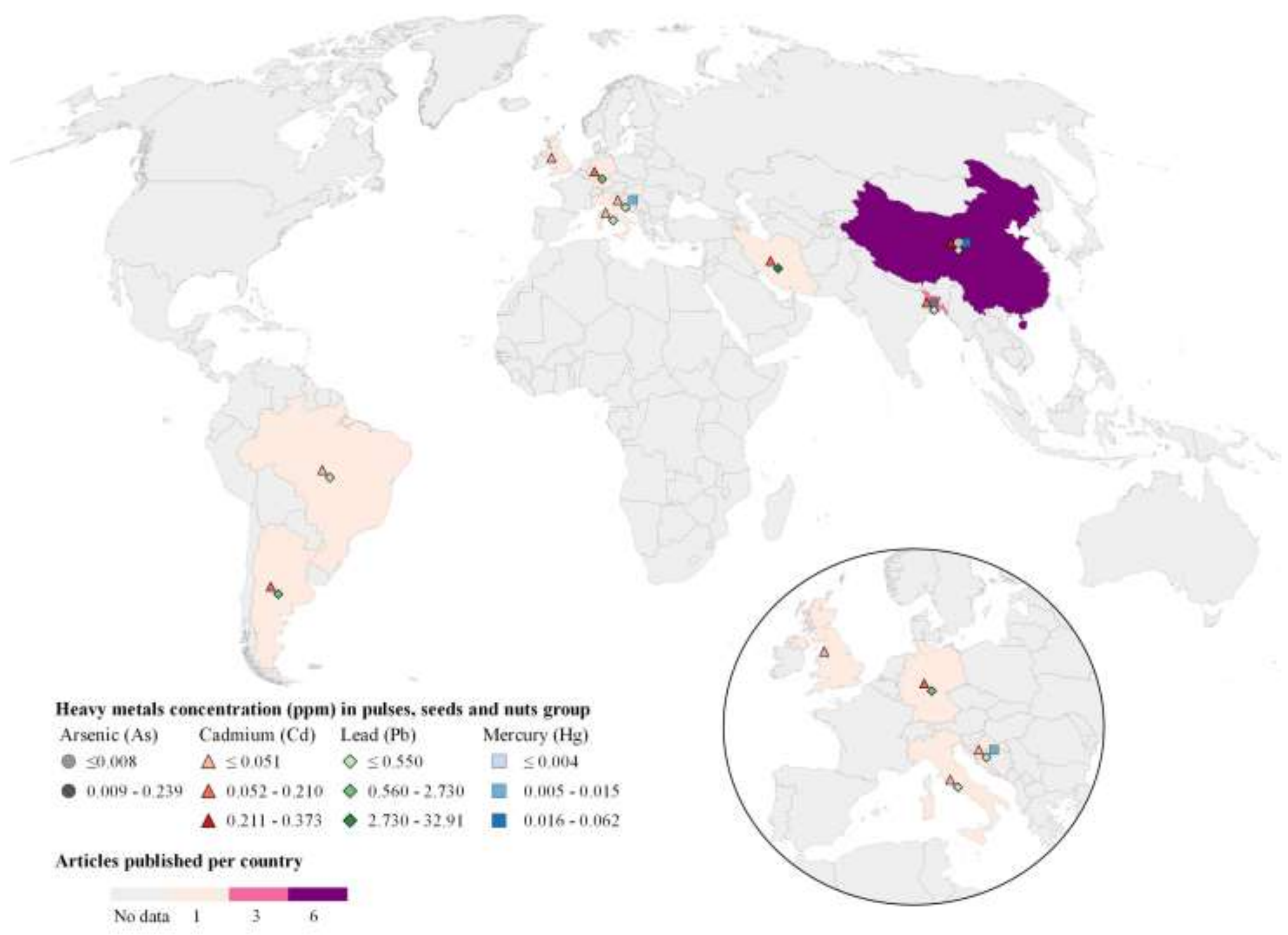

Map 6. Heavy metals concentration in Pulses, seeds, and nuts food group by country

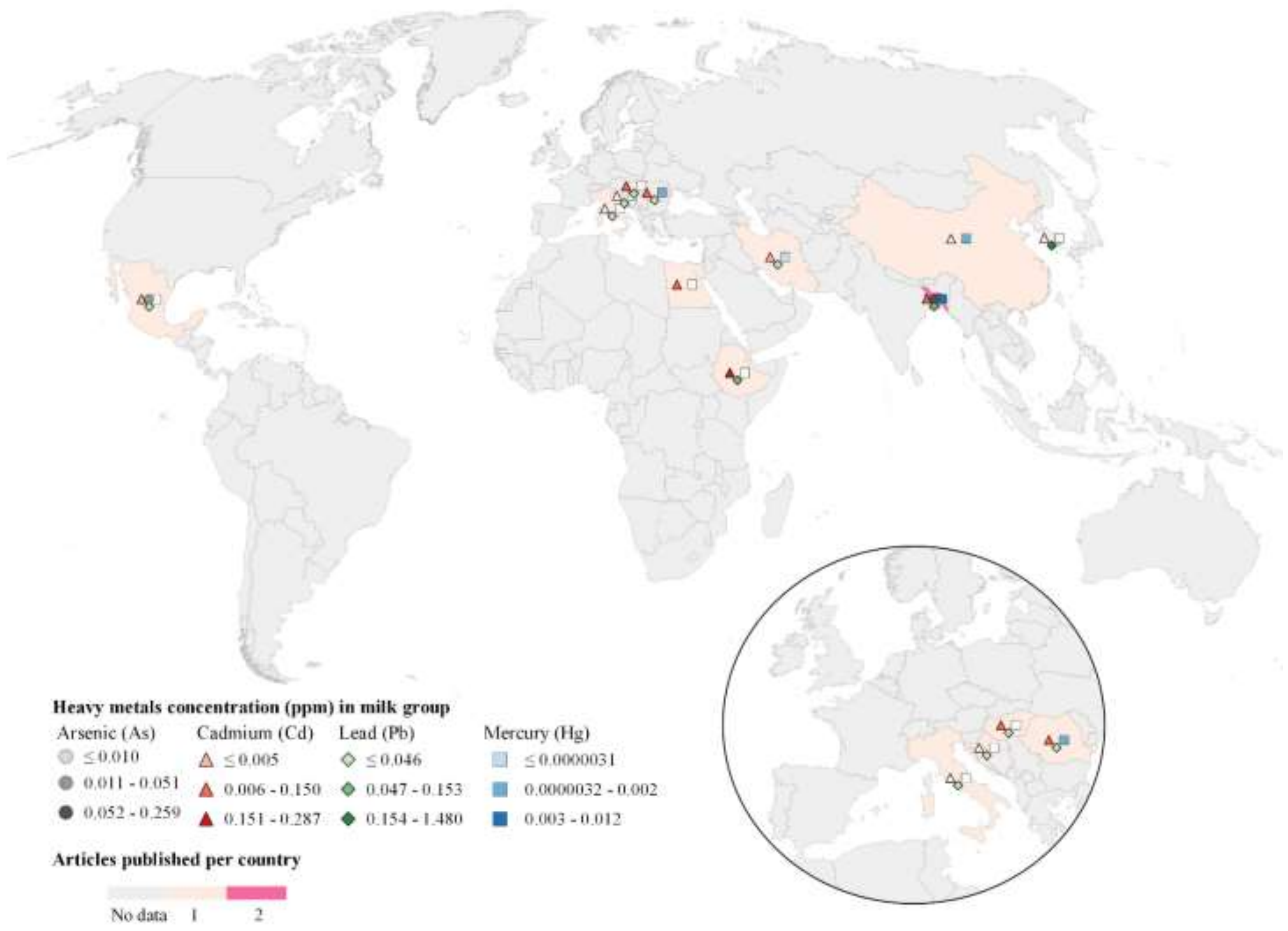

Map 7. Heavy metals concentration in Milk food group by country

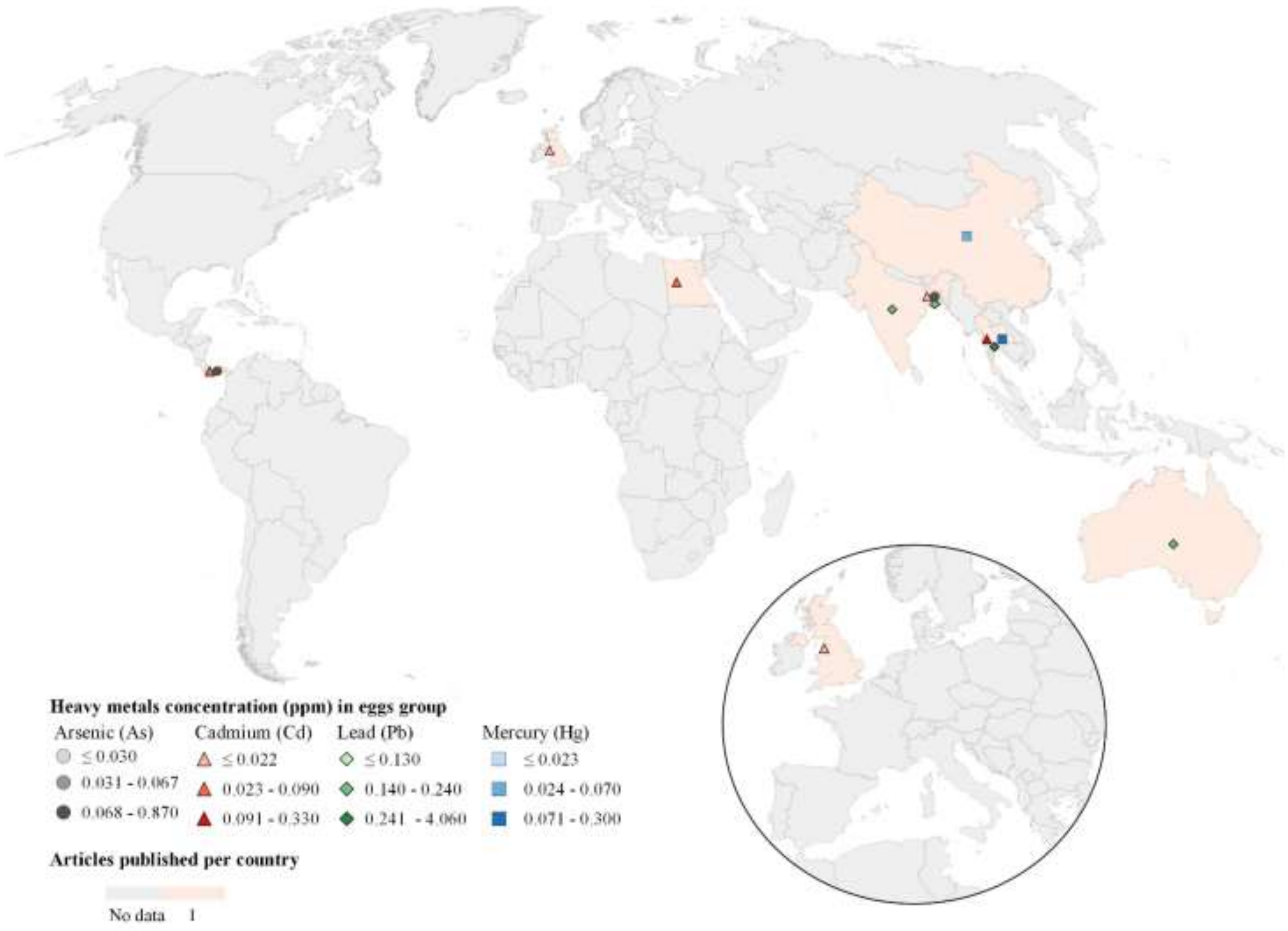

Map 8. Heavy metals content concentration in Eggs food group by country

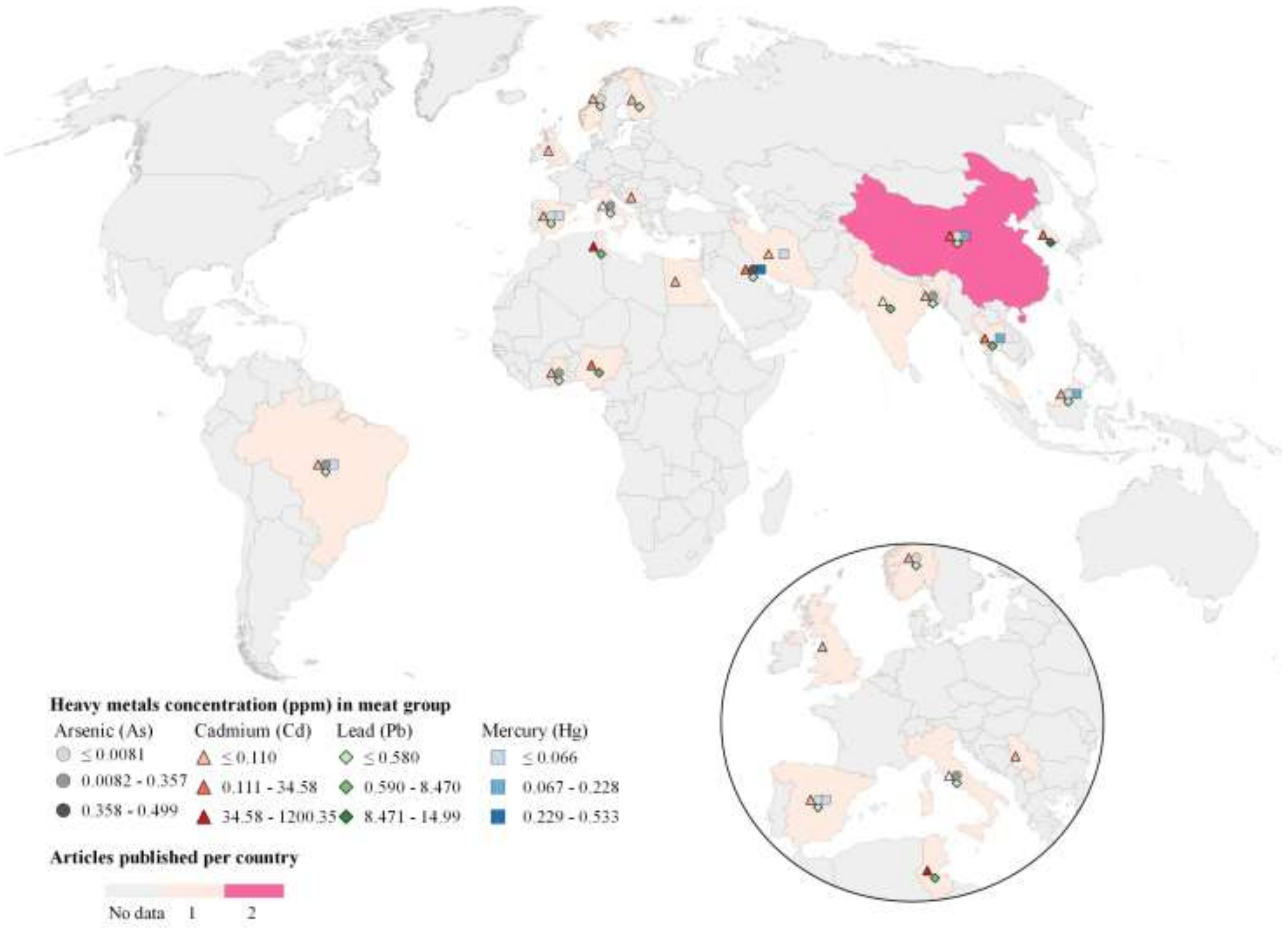

Map 9. Heavy metals content concentration in Meat food group by country

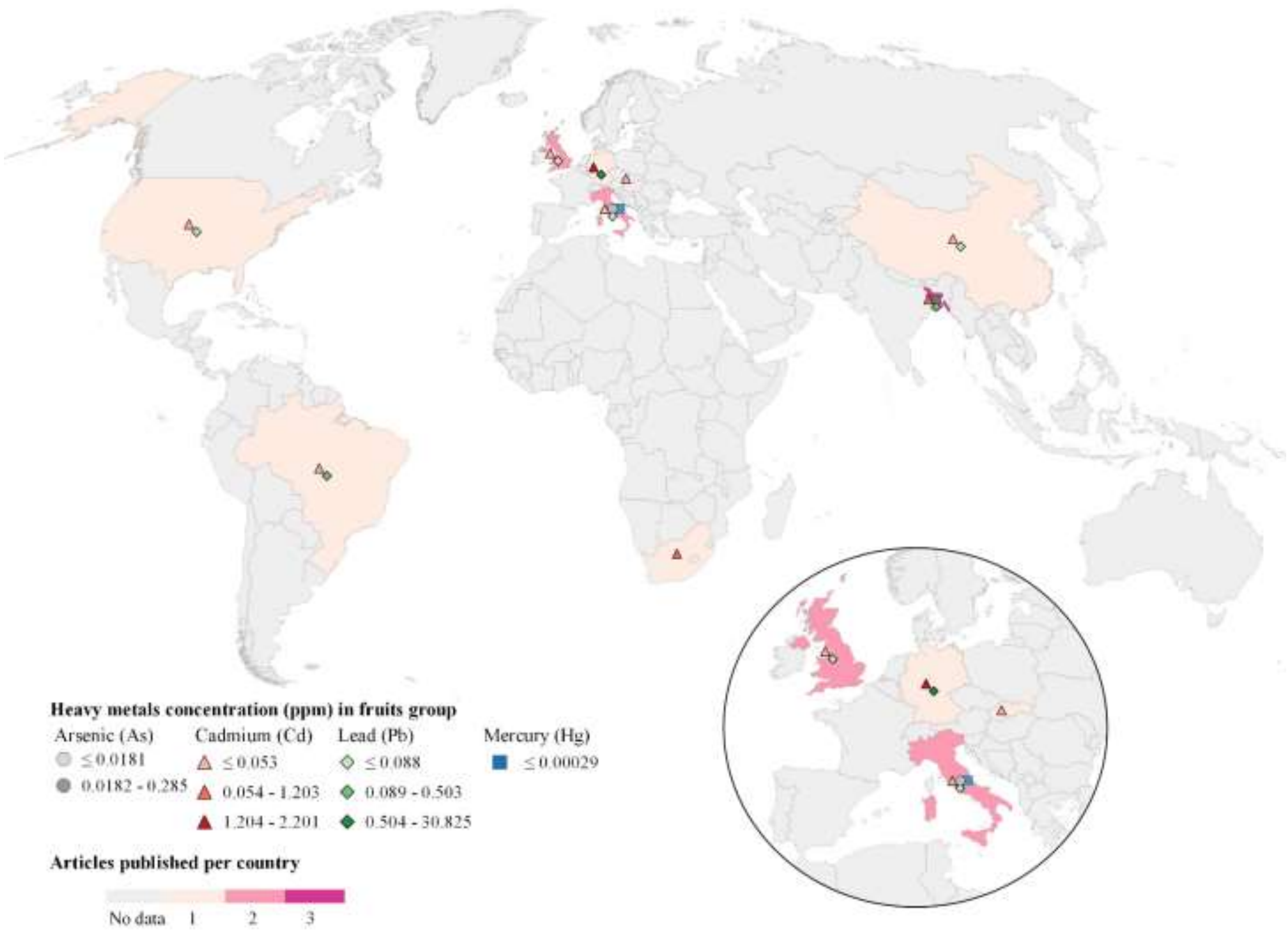

Map 10. Heavy metals concentration in Fruits food group by country

Source: Authors elaboration accordingly to the studies information that were included in this Scoping Review [1-152].

References

1. Malavolti, M.; Fairweather-tait, S.J.; Malagoli, C.; Vescovi, L. Lead Exposure in an Italian Population : Food Content , Dietary Intake and Risk Assessment. *Food Res. Int.* **2020**, *137*, 109370, doi:10.1016/j.foodres.2020.109370.
2. Năstăsescu, V.; Mititelu, M.; Goumenou, M.; Docea, A.O.; Renieri, E.; Udeanu, D.I.; Oprea, E.; Arsene, A.L.; Dinu-Pîrvu, C.E.; Ghica, M. Heavy Metal and Pesticide Levels in Dairy Products: Evaluation of Human Health Risk. *Food Chem. Toxicol.* **2020**, *146*, 111844, doi:10.1016/j.fct.2020.111844.
3. Custódio, F.B.; Andrade, A.M.G.F.; Guidi, L.R.; Leal, C.A.G.; Gloria, M.B.A. Total Mercury in Commercial Fishes and Estimation of Brazilian Dietary Exposure to Methylmercury. *J. Trace Elem. Med. Biol.* **2020**, *62*, 126641, doi:10.1016/j.jtemb.2020.126641.
4. Di Bella, G.; Bua, G.D.; Fede, M.R.; Mottese, A.F.; Potortì, A.G.; Cicero, N.; Benameur, Q.; Dugo, G.; Lo Turco, V. Potentially Toxic Elements in Xiphias Gladius from Mediterranean Sea and Risks Related to Human Consumption. *Mar. Pollut. Bull.* **2020**, *159*, 111512, doi:10.1016/j.marpolbul.2020.111512.
5. Djedjibegovic, J.; Marjanovic, A.; Tahirovic, D.; Caklovica, K.; Turalic, A.; Lugusic, A.; Omeragic, E.; Sober, M.; Caklovica, F. Heavy Metals in Commercial Fish and Seafood Products and Risk Assessment in Adult Population in Bosnia and Herzegovina. *Sci. Rep.* **2020**, *10*, 13238, doi:10.1038/s41598-020-70205-9.
6. Di Bella, C.; Traina, A.; Giosuè, C.; Carpintieri, D.; Lo Dico, G.M.; Bellante, A.; Del Core, M.; Falco, F.; Gherardi, S.; Uccello, M.M.; et al. Heavy Metals and PAHs in Meat, Milk, and Seafood From Augusta Area (Southern Italy): Contamination Levels, Dietary Intake, and Human Exposure Assessment. *Front. Public Heal.* **2020**, *8*, doi:10.3389/fpubh.2020.00273.
7. Liu, L.; Han, J.; Xu, X.; Xu, Z.; Abeysinghe, K.S.; Atapattu, A.J.; De Silva, P.M.C.S.; Lu, Q.; Qiu, G. Dietary Exposure Assessment of Cadmium, Arsenic, and Lead in Market Rice from Sri Lanka. *Environ. Sci. Pollut. Res.* **2020**, *27*, 42704–42712, doi:10.1007/s11356-020-10209-0.
8. Kukusamude, C.; Sricharoen, P.; Limchoowong, N.; Kongsri, S. Heavy Metals and Probabilistic Risk Assessment via Rice Consumption in Thailand. *Food Chem.* **2021**, *334*, 127402, doi:10.1016/j.foodchem.2020.127402.
9. Milatou, N.; Dassenakis, M.; Megalofonou, P. Mercury Concentrations in Reared Atlantic Bluefin Tuna and Risk Assessment for the Consumers: To Eat or Not to Eat? Food Chem. 2020, 331, 127267, doi:10.1016/j.foodchem.2020.127267.
10. Kosker, A.R. Metal and Fatty Acid Levels of Some Commercially Important Marine Species from the Northeastern Mediterranean: Benefits and Health Risk Estimation. *Environ. Monit. Assess.* **2020**, *192*, 358, doi:10.1007/s10661-020-08287-1.
11. Branciari, R.; Franceschini, R.; Roila, R.; Valiani, A.; Pecorelli, I.; Piersanti, A.; Haouet, N.; Framboas, M.; Ranucci, D. Nutritional Value and Contaminant Risk Assessment of Some Commercially Important Fishes and Crawfish of Lake Trasimeno, Italy. *Int. J. Environ. Res. Public Health* **2020**, *17*, 2545, doi:10.3390/ijerph17072545.
12. Yu, X.; Khan, S.; Khan, A.; Tang, Y.; Nunes, L.M.; Yan, J.; Ye, X.; Li, G. Methylmercury Concentrations in Seafood Collected from Zhoushan Islands, Zhejiang, China, and Their Potential Health Risk for the Fishing Community. *Environ. Int.* **2020**, *137*, 105420, doi:10.1016/j.envint.2019.105420.
13. Abd-Elghany, S.M.; Mohammed, M.A.; Abdelkhalek, A.; Saad, F.S.S.; Sallam, K.I. Health Risk Assessment of Exposure to Heavy Metals from Sheep Meat and Offal in Kuwait. *J. Food Prot.* **2020**, *83*, 503–510, doi:10.4315/0362-028X.JFP-19-265.
14. Shahriar, S.; Rahman, M.M.; Naidu, R. Geographical Variation of Cadmium in Commercial Rice Brands in Bangladesh: Human Health Risk Assessment. *Sci. Total Environ.* **2020**, *716*, 137049, doi:10.1016/j.scitotenv.2020.137049.
15. Li, L.; Feng, H.; Wei, J. Toxic Element (As and Hg) Content and Health Risk Assessment of Commercially Available Rice for Residents in Beijing Based on Their Dietary Consumption. *Environ. Sci. Pollut. Res.* **2020**, *27*, 13205–13214, doi:10.1007/s11356-020-07790-9.
16. Heshmati, A.; Mehri, F.; Karami-Momtaz, J.; Khaneghah, A.M. Concentration and Risk Assessment of Potentially Toxic Elements, Lead and Cadmium, in Vegetables and Cereals Consumed in Western Iran. *J. Food Prot.* **2020**, *83*, 101–107, doi:10.4315/0362-028X.JFP-19-312.
17. Malvandi, H.; Alahabadi, A. Evaluation of Potential Human Health Risk Due to the Exposure to Mercury via Fish Consumption of Alosa Spp. from the Southern Caspian Sea. *Mar. Pollut. Bull.* **2019**, *143*, 66–71, doi:10.1016/j.marpolbul.2019.04.042.
18. Gong, Y.; Chai, M.; Ding, H.; Shi, C.; Wang, Y.; Li, R. Bioaccumulation and Human Health Risk of Shellfish Contamination to Heavy Metals and As in Most Rapid Urbanized Shenzhen, China. *Environ. Sci. Pollut. Res.* **2020**, *27*, 2096–2106, doi:10.1007/s11356-019-06580-2.
19. Djermanovic, M.; Baralic, I.; Pejic, S. Total Mercury Levels in Commercial Fish in Market of the Republic of Srpska, Bosnia and Herzegovina. *Biol. Trace Elem. Res.* **2020**, *194*, 545–551, doi:10.1007/s12011-019-01805-4.
20. Aendo, P.; Thongyuan, S.; Songserm, T.; Tulayakul, P. Carcinogenic and Non-Carcinogenic Risk Assessment of Heavy Metals Contamination in Duck Eggs and Meat as a Warning Scenario in Thailand. *Sci. Total Environ.* **2019**, *689*, 215–222, doi:10.1016/j.scitotenv.2019.06.414.
21. Yang, W.; Wang, D.; Wang, M.; Zhou, F.; Huang, J.; Xue, M.; Dinh, Q.T.; Liang, D. Heavy Metals and Associated Health Risk of Wheat Grain in a Traditional Cultivation Area of Baoji, Shaanxi, China. *Environ. Monit. Assess.* **2019**, *191*, 428, doi:10.1007/s10661-019-7534-9.
22. Hwang, D.-W.; Shim, K.; Lee, C. Il Concentrations and Risk Assessment of Heavy Metals in Tissues of Walleye Pollock (Gadus Chalcogrammus) Captured from the Northeastern Coast of Korea. *J. Food Prot.* **2019**, *82*, 903–909, doi:10.4315/0362-028X.JFP-18-379.
23. Annibaldi, A.; Truzzi, C.; Carnevali, O.; Pignalosa, P.; Api, M.; Scarponi, G.; Illuminati, S. Determination of Hg in Farmed and Wild Atlantic Bluefin Tuna (Thunnus Thynnus L.) Muscle. *Molecules* **2019**, *24*, 1273, doi:10.3390/molecules24071273.
24. Darwish, W.S.; Chiba, H.; Elhelaly, A.E.; Hui, S.-P. Estimation of Cadmium Content in Egyptian Foodstuffs: Health Risk Assessment, Biological Responses of Human HepG2 Cells to Food-Relevant Concentrations of Cadmium, and Protection Trials Using Rosmarinic and Ascorbic Acids. *Environ. Sci. Pollut. Res.* **2019**, *26*, 15443–15457, doi:10.1007/s11356-019-04852-5.
25. Kim, S.W.; Han, S.J.; Kim, Y.; Jun, J.W.; Giri, S.S.; Chi, C.; Yun, S.; Kim, H.J.; Kim, S.G.; Kang, J.W.; et al. Heavy Metal Accumulation in and Food Safety of Shark Meat from Jeju Island, Republic of Korea. *PLoS One* **2019**, *14*, e0212410, doi:10.1371/journal.pone.0212410.
26. Kato, L.S.; De Nadai Fernandes, E.A.; Raab, A.; Bacchi, M.A.; Feldmann, J. Arsenic and Cadmium Contents in Brazilian Rice from Different Origins Can Vary More than Two Orders of Magnitude. *Food Chem.* **2019**, *286*, 644–650, doi:10.1016/j.foodchem.2019.02.043.
27. Lin, H.; Santa-Rios, A.; Barst, B.D.; Basu, N.; Bayen, S. Occurrence and Bioaccessibility of Mercury in Commercial Rice Samples in Montreal (Canada). *Food Chem. Toxicol.* **2019**, *126*, 72–78, doi:10.1016/j.fct.2019.02.006.
28. Zeinali, T.; Salmani, F.; Naseri, K. Dietary Intake of Cadmium, Chromium, Copper, Nickel, and Lead through the Consumption of Meat, Liver, and Kidney and Assessment of Human Health Risk in Birjand, Southeast of Iran. *Biol. Trace Elem. Res.* **2019**, *191*, 338–347, doi:10.1007/s12011-019-1637-6.
29. Rahman, M.; Islam, M.A. Concentrations and Health Risk Assessment of Trace Elements in Cereals, Fruits, and Vegetables of Bangladesh. *Biol. Trace Elem. Res.* **2019**, *191*, 243–253, doi:10.1007/s12011-018-1596-3.
30. Paz, S.; Rubio, C.; Frías, I.; Gutiérrez, Á.J.; González-Weller, D.; Martín, V.; Revert, C.; Hardisson, A. Toxic Metals (Al, Cd, Pb and Hg) in the Most Consumed Edible Seaweeds in Europe. *Chemosphere* **2019**, *218*, 879–884, doi:10.1016/j.chemosphere.2018.11.165.
31. Traina, A.; Bono, G.; Bonsignore, M.; Falco, F.; Giuga, M.; Quinci, E.M.; Vitale, S.; Sprovieri, M. Heavy Metals Concentrations in Some Commercially Key Species from Sicilian Coasts (Mediterranean Sea): Potential Human Health Risk Estimation. *Ecotoxicol. Environ. Saf.* **2019**, *168*, 466–478, doi:10.1016/j.ecoenv.2018.10.056.
32. Zariff, R.Z.; Pumpa, L.; Simon, D.L.; Lewis, C. Locally Produced Chicken Eggs—a Source of Dietary Lead for an Australian Community Living with an Active Lead Smelter? *J. Expo. Sci. Environ. Epidemiol.* **2019**, *29*, 688–696, doi:10.1038/s41370-018-0082-5.
33. Rahmdel, S.; Rezaei, M.; Ekhlasi, J.; Zarei, S.H.; Akhlaghi, M.; Abdollahzadeh, S.M.; Sefidkar, R.; Mazloomi, S.M. Heavy Metals (Pb, Cd, Cu, Zn, Ni, Co) in Leafy Vegetables Collected from Production Sites: Their Potential Health Risk to the General Population in Shiraz, Iran. *Environ. Monit. Assess.* **2018**, *190*, 650, doi:10.1007/s10661-018-7042-3.
34. Zamora-Arellano, N.; Betancourt-Lozano, M.; Ilizaliturri-Hernández, C.; García-Hernández, J.; Jara-Marini, M.; Chávez-Sánchez, C.; Ruelas-Inzunza, J.R. Mercury Levels and Risk Implications Through Fish Consumption on the Sinaloa Coasts (Gulf of California, Northwest Mexico). *Risk Anal.* **2018**, *38*, 2646–2658, doi:10.1111/risa.13185.
35. Fakhri, Y.; Mousavi Khaneghah, A.; Conti, G.O.; Ferrante, M.; Khezri, A.; Darvishi, A.; Ahmadi, M.; Hasanzadeh, V.; Rahimizadeh, A.; Keramati, H.; et al. Probabilistic Risk Assessment (Monte Carlo Simulation Method) of Pb and Cd in the Onion Bulb (Allium Cepa) and Soil of Iran. *Environ. Sci. Pollut. Res.* **2018**, *25*, 30894–30906, doi:10.1007/s11356-018-3081-0.
36. Costa, R.G.; Araújo, C.F. da S.; Ferreol Bah, A.H.; Junior, E.A.G.; Rodrigues, Y.J. de M.; Menezes-Filho, J.A. Lead in Mangrove Root Crab (*Goniopsis Cruentata*) and Risk Assessment Due to Exposure for Estuarine Villagers. *Food Addit. Contam. Part B* **2018**, *11*, 293–301, doi:10.1080/19393210.2018.1504824.
37. Lehel, J.; Bartha, A.; Dankó, D.; Lányi, K.; Laczay, P. Heavy Metals in Seafood Purchased from a Fishery Market in Hungary. *Food Addit. Contam. Part B* **2018**, *11*, 302–308, doi:10.1080/19393210.2018.1505781.
38. Huo, J.; Huang, Z.; Li, R.; Song, Y.; Lan, Z.; Ma, S.; Wu, Y.; Chen, J.; Zhang, L. Dietary Cadmium Exposure Assessment in Rural Areas of Southwest China. *PLoS One* **2018**, *13*, e0201454, doi:10.1371/journal.pone.0201454.
39. Chen, H.; Yang, X.; Wang, P.; Wang, Z.; Li, M.; Zhao, F.-J. Dietary Cadmium Intake from Rice and Vegetables and Potential Health Risk: A Case Study in Xiangtan, Southern China. *Sci. Total Environ.* **2018**, *639*, 271–277, doi:10.1016/j.scitotenv.2018.05.050.
40. Bi, C.; Zhou, Y.; Chen, Z.; Jia, J.; Bao, X. Heavy Metals and Lead Isotopes in Soils, Road Dust and Leafy Vegetables and Health Risks via Vegetable Consumption in the Industrial Areas of Shanghai, China. *Sci. Total Environ.* **2018**, *619–620*, 1349–1357, doi:10.1016/j.scitotenv.2017.11.177.
41. Djahed, B.; Taghavi, M.; Farzadkia, M.; Norzaee, S.; Miri, M. Stochastic Exposure and Health Risk Assessment of Rice Contamination to the Heavy Metals in the Market of Iranshahr, Iran. *Food Chem. Toxicol.* **2018**, *115*, 405–412, doi:10.1016/j.fct.2018.03.040.
42. Chen, H.; Tang, Z.; Wang, P.; Zhao, F.-J. Geographical Variations of Cadmium and Arsenic Concentrations and Arsenic Speciation in Chinese Rice. *Environ. Pollut.* **2018**, *238*, 482–490, doi:10.1016/j.envpol.2018.03.048.
43. Mallory, M.L.; O'Driscoll, N.J.; Klapstein, S.; Varela, J.L.; Ceapa, C.; Stokesbury, M.J. Methylmercury in Tissues of Atlantic Sturgeon (Acipenser Oxyrhynchus) from the Saint John River, New Brunswick, Canada. *Mar. Pollut. Bull.* **2018**, *126*, 250–254, doi:10.1016/j.marpolbul.2017.11.024.
44. Ding, Z.; Li, Y.; Sun, Q.; Zhang, H. Trace Elements in Soils and Selected Agricultural Plants in the Tongling Mining Area of China. *Int. J. Environ. Res. Public Health* **2018**, *15*, 202, doi:10.3390/ijerph15020202.
45. Giri, S.; Singh, A.K. Heavy Metals in Eggs and Chicken and the Associated Human Health Risk Assessment in the Mining Areas of Singhbhum Copper Belt, India. *Arch. Environ. Occup. Health* **2019**, *74*, 161–170,

doi:10.1080/19338244.2017.1407284.
46. Hoang, V.A.T.; Sakamoto, M.; Yamamoto, M. Mercury and Selenium Levels, and Their Molar Ratios in Several Species of Commercial Shrimp in Japan Regarding the Health Risk of Methylmercury Exposure. *J. Toxicol. Sci.* **2017**, *42*, 509–517, doi:10.2131/jts.42.509.
47. Varol, M.; Kaya, G.K.; Alp, A. Heavy Metal and Arsenic Concentrations in Rainbow Trout (Oncorhynchus Mykiss) Farmed in a Dam Reservoir on the Firat (Euphrates) River: Risk-Based Consumption Advisories. *Sci. Total Environ.* **2017**, *599–600*, 1288–1296, doi:10.1016/j.scitotenv.2017.05.052.
48. Cheng, J.; Zhang, X.; Tang, Z.; Yang, Y.; Nie, Z.; Huang, Q. Concentrations and Human Health Implications of Heavy Metals in Market Foods from a Chinese Coal-Mining City. *Environ. Toxicol. Pharmacol.* **2017**, *50*, 37–44, doi:10.1016/j.etap.2017.01.011.
49. Stančić, Z.; Vujević, D.; Gomaz, A.; Bogdan, S.; Vincek, D. Detection of Heavy Metals in Common Vegetables at Varaždin City Market, Croatia. *Arch. Ind. Hyg. Toxicol.* **2016**, *67*, 340–350, doi:10.1515/aiht-2016-67-2823.
50. Roya, A.Q.; Ali, M.S. Heavy Metals in Rice Samples on the Torbat-Heidarieh Market, Iran. *Food Addit. Contam. Part B* **2017**, *10*, 59–63, doi:10.1080/19393210.2016.1247918.
51. García, M.Á.; Núñez, R.; Alonso, J.; Melgar, M.J. Total Mercury in Fresh and Processed Tuna Marketed in Galicia (NW Spain) in Relation to Dietary Exposure. *Environ. Sci. Pollut. Res.* **2016**, *23*, 24960–24969, doi:10.1007/s11356-016-7634-9.
52. Xie, L.H.; Tang, S.Q.; Wei, X.J.; Shao, G.N.; Jiao, G.A.; Sheng, Z.H.; Luo, J.; Hu, P.S. The Cadmium and Lead Content of the Grain Produced by Leading Chinese Rice Cultivars. *Food Chem.* **2017**, *217*, 217–224, doi:10.1016/j.foodchem.2016.08.099.
53. Adel, M.; Oliveri Conti, G.; Dadar, M.; Mahjoub, M.; Copat, C.; Ferrante, M. Heavy Metal Concentrations in Edible Muscle of Whitecheek Shark, Carcharhinus Dussumieri (Elasmobranchii, Chondrichthyes) from the Persian Gulf: A Food Safety Issue. *Food Chem. Toxicol.* **2016**, *97*, 135–140, doi:10.1016/j.fct.2016.09.002.
54. Ross, D.A.; Guzmán, H.M.; Van Hinsberg, V.J.; Potvin, C. Metal Contents of Marine Turtle Eggs (Chelonia Mydas; Lepidochelys Olivacea) from the Tropical Eastern Pacific and the Implications for Human Health. *J. Environ. Sci. Heal. Part B* **2016**, *51*, 675–687, doi:10.1080/03601234.2016.1191888.
55. López-Alonso, M.; Miranda, M.; Benedito, J.L.; Pereira, V.; García-Vaquero, M. Essential and Toxic Trace Element Concentrations in Different Commercial Veal Cuts in Spain. *Meat Sci.* **2016**, *121*, 47–52, doi:10.1016/j.meatsci.2016.05.013.
56. Avci, H.; Deveci, T. Assessment of Trace Element Concentrations in Soil and Plants from Cropland Irrigated with Wastewater. *Ecotoxicol. Environ. Saf.* **2013**, *98*, 283–291, doi:10.1016/j.ecoenv.2013.08.013.
57. Islam, M.S.; Ahmed, M.K.; Habibullah-Al-Mamun, M.; Masunaga, S. Assessment of Trace Metals in Foodstuffs Grown around the Vicinity of Industries in Bangladesh. *J. Food Compos. Anal.* **2015**, *42*, 8–15, doi:10.1016/j.jfca.2014.12.031.
58. Djedjibegovic, J.; Larssen, T.; Skrbo, A.; Marjanović, A.; Sober, M. Contents of Cadmium, Copper, Mercury and Lead in Fish from the Neretva River (Bosnia and Herzegovina) Determined by Inductively Coupled Plasma Mass Spectrometry (ICP-MS). *Food Chem.* **2012**, *131*, 469–476, doi:10.1016/j.foodchem.2011.09.009.
59. Derakhshan, Z.; Mahvi, A.H.; Faramarzian, M.; Dehghani, M.; Salari, M.; Fakhri, Y.; Afsharnia, M.; Hosseini, M.S.; Marzban, A.; Taghavi, M. Data on Heavy Metal Concentration in Common Carp Fish Consumed in Shiraz, Iran. *Data Br.* **2018**, *21*, 1890–1894, doi:10.1016/j.dib.2018.11.029.
60. Wu, X.-Y.; Yang, Y.-F. Heavy Metal (Pb, Co, Cd, Cr, Cu, Fe, Mn and Zn) Concentrations in Harvest-Size White Shrimp Litopenaeus Vannamei Tissues from Aquaculture and Wild Source. *J. Food Compos. Anal.* **2011**, *24*, 62–65, doi:10.1016/j.jfca.2010.03.030.
61. Peng, M.; Zhao, C.; Ma, H.; Yang, Z.; Yang, K.; Liu, F.; Li, K.; Yang, Z.; Tang, S.; Guo, F.; et al. Heavy Metal and Pb Isotopic Compositions of Soil and Maize from a Major Agricultural Area in Northeast China: Contamination Assessment and Source Apportionment. *J. Geochemical Explor.* **2020**, *208*, 106403, doi:10.1016/j.gexplo.2019.106403.
62. Keshavarzi, B.; Hassanaghaei, M.; Moore, F.; Rastegari Mehr, M.; Soltanian, S.; Lahijanzadeh, A.R.; Sorooshian, A. Heavy Metal Contamination and Health Risk Assessment in Three Commercial Fish Species in the Persian Gulf. *Mar. Pollut. Bull.* **2018**, *129*, 245–252, doi:10.1016/j.marpolbul.2018.02.032.
63. Rabiul Islam, G.M.; Habib, M.R.; Waid, J.L.; Rahman, M.S.; Kabir, J.; Akter, S.; Jolly, Y.N. Heavy Metal Contamination of Freshwater Prawn (Macrobrachium Rosenbergii) and Prawn Feed in Bangladesh: A Market-Based Study to Highlight Probable Health Risks. *Chemosphere* **2017**, *170*, 282–289, doi:10.1016/j.chemosphere.2016.11.163.
64. Antoniadis, V.; Golia, E.E.; Liu, Y.-T.; Wang, S.-L.; Shaheen, S.M.; Rinklebe, J. Soil and Maize Contamination by Trace Elements and Associated Health Risk Assessment in the Industrial Area of Volos, Greece. *Environ. Int.* **2019**, *124*, 79–88, doi:10.1016/j.envint.2018.12.053.
65. Yang, B.; Zhang, C.; Zhang, X.; Wang, G.; Li, L.; Geng, H.; Liu, Y.; Nie, C. Survey of Aflatoxin B1 and Heavy Metal Contamination in Peanut and Peanut Soil in China during 2017–2018. *Food Control* **2020**, *118*, 107372, doi:10.1016/j.foodcont.2020.107372.
66. Naghipour, D.; Chenari, M.A.; Taheri, N.; Naghipour, F.; Mehrabian, F.; Attarchi, M.S.; Jaafari, J.; Roubakhsh, E. The Concentration Data of Heavy Metals in Vegetables of Guilan Province, Iran. *Data Br.* **2018**, *21*, 1704–1708, doi:10.1016/j.dib.2018.10.114.
67. de Souza Araújo, D.F.; da Silva, A.M.R.B.; de Andrade Lima, L.L.; da Silva Vasconcelos, M.A.; Andrade, S.A.C.; Asfora Sarubbo, L. The Concentration of Minerals and Physicochemical Contaminants in Conventional and Organic Vegetables. *Food Control* **2014**, *44*, 242–248, doi:10.1016/j.foodcont.2014.04.005.
68. Anandkumar, A.; Li, J.; Prabakaran, K.; Xi Jia, Z.; Leng, Z.; Nagarajan, R.; Du, D. Accumulation of Toxic Elements in an Invasive Crayfish Species (Procambarus Clarkii) and Its Health Risk Assessment to Humans. *J. Food Compos. Anal.* **2020**, *88*, 103449, doi:10.1016/j.jfca.2020.103449.
69. Krejčová, A.; Návesník, J.; Jičínská, J.; Černohorský, T. An Elemental Analysis of Conventionally, Organically and Self-Grown Carrots. *Food Chem.* **2016**, *192*, 242–249, doi:10.1016/j.foodchem.2015.07.008.
70. Nikolic, N.; Borisev, M.; Pajevic, S.; Arsenov, D.; Zupunski, M. Comparative Assessment of Mineral Elements and Heavy Metals Accumulation in Vegetable Species. *Food Feed Res.* **2014**, *41*, 115–123, doi:10.5937/FFR1402115N.
71. Bat, L.; Öztekin, A.; Arici, E.; Şahin, F. Health Risk Assessment: Heavy Metals in Fish from the Southern Black Sea. *Foods Raw Mater.* **2020**, *8*, 115–124, doi:10.21603/2308-4057-2020-1-115-124.
72. Taghipour, H.; Mosaferi, M. Heavy Metals in the Vegetables Collected from Production Sites. *Heal. Promot Perspect* **2013**, *3*, 185–193.
73. Guerra, F.; Trevizam, A.R.; Muraoka, T.; Marcante, N.C.; Canniatti-Brazaca, S.G. Heavy Metals in Vegetables and Potential Risk for Human Health. *Sci. Agric.* **2012**, *69*, 54–60, doi:10.1590/S0103-90162012000100008.
74. Melai, V.; Giovannini, A.; Chiumiento, F.; Bellocci, M.; Migliorati, G. Occurrence of Metals in Vegetables and Fruits from Areas near Landfill in Southern Italy and Implications for Human Exposure. *Int. J. Food Contam.* **2018**, *5*, 8, doi:10.1186/s40550-018-0070-5.
75. Shaheen, N.; Irfan, N.M.; Khan, I.N.; Islam, S.; Islam, M.S.; Ahmed, M.K. Presence of Heavy Metals in Fruits and Vegetables: Health Risk Implications in Bangladesh. *Chemosphere* **2016**, *152*, 431–438, doi:https://doi.org/10.1016/j.chemosphere.2016.02.060.
76. Zhou, H.; Yang, W.-T.; Zhou, X.; Liu, L.; Gu, J.-F.; Wang, W.-L.; Zou, J.-L.; Tian, T.; Peng, P.-Q.; Liao, B.-H. Accumulation of Heavy Metals in Vegetable Species Planted in Contaminated Soils and the Health Risk Assessment. *Int. J. Environ. Res. Public Health* **2016**, *13*, 289, doi:10.3390/ijerph13030289.
77. Bilandžić, N.; Sedak, M.; Čalopek, B.; Luburić, Đ.B.; Solomun Kolanović, B.; Varenina, I.; Đokić, M.; Kmetič, I.; Murati, T. Lead Concentrations in Raw Cow and Goat Milk Collected in Rural Areas of Croatia from 2010 to 2014. *Bull. Environ. Contam. Toxicol.* **2016**, *96*, 645–649, doi:10.1007/s00128-016-1749-z.
78. Pan, X.-D.; Wu, P.-G.; Jiang, X.-G. Levels and Potential Health Risk of Heavy Metals in Marketed Vegetables in Zhejiang, China. *Sci. Rep.* **2016**, *6*, 20317, doi:10.1038/srep20317.
79. Ariano, A.; Lo Voi, A.; D'Ambola; Marrone, R.; Cacace, D.; Severino, L. Levels of Cadmium in White and Brown Meat of Warty Crab (Eriphia Verrucosa). *J. Food Prot.* **2015**, *78*, 2253–2256, doi:10.4315/0362-028X.JFP-15-214.
80. Kim, D.-G.; Kim, M.; Shin, J.Y.; Son, S.-W. Cadmium and Lead in Animal Tissue (Muscle, Liver and Kidney), Cow Milk and Dairy Products in Korea. *Food Addit. Contam. Part B* **2016**, *9*, 33–37, doi:10.1080/19393210.2015.1114032.
81. Arroyo-Abad, U.; Pfeifer, M.; Mothes, S.; Stärk, H.-J.; Piechotta, C.; Mattusch, J.; Reemtsma, T. Determination of Moderately Polar Arsenolipids and Mercury Speciation in Freshwater Fish of the River Elbe (Saxony, Germany). *Environ. Pollut.* **2016**, *208*, 458–466, doi:10.1016/j.envpol.2015.10.015.
82. Pirsaheb, M.; Fattahi, N.; Sharafi, K.; Khamotian, R.; Atafar, Z. Essential and Toxic Heavy Metals in Cereals and Agricultural Products Marketed in Kermanshah, Iran, and Human Health Risk Assessment. *Food Addit. Contam. Part B* **2016**, *9*, 15–20, doi:10.1080/19393210.2015.1099570.
83. Rahayu, R.N.; Irawan, B.; Soegianto, A. Concentration of Mercury in Cockles (Anadara Granosa and A. Antiquata) Harvested from Estuaries of Western Lombok, Indonesia, and Potential Risks to Human Health. *Bull. Environ. Contam. Toxicol.* **2016**, *96*, 20–24, doi:10.1007/s00128-015-1672-8.
84. Molognoni, L.; Vitali, L.; Ploêncio, L.A.; Santos, J.N.; Daguer, H. Determining the Arsenic, Cadmium, Lead, Copper and Chromium Contents by Atomic Absorption Spectrometry in *Pangasius* Fillets from Vietnam. *J. Sci. Food Agric.* **2016**, *96*, 3109–3113, doi:10.1002/jsfa.7486.
85. Musilova, J.; Bystricka, J.; Lachman, J.; Harangozo, L.; Trebichalsky, P.; Volnova, B. Potatoes – A Crop Resistant against Input of Heavy Metals from the Metallicaly Contaminated Soil. *Int. J. Phytoremediation* **2016**, *18*, 547–552, doi:10.1080/15226514.2015.1086303.
86. Esposito, M.; Picazio, G.; Serpe, P.; Lambiase, S.; Cerino, P. Content of Cadmium and Lead in Vegetables and Fruits Grown in the Campania Region of Italy. *J. Food Prot.* **2015**, *78*, 1760–1765, doi:10.4315/0362-028X.JFP-15-072.
87. Tang, W.; Cheng, J.; Zhao, W.; Wang, W. Mercury Levels and Estimated Total Daily Intakes for Children and Adults from an Electronic Waste Recycling Area in Taizhou, China: Key Role of Rice and Fish Consumption. *J. Environ. Sci.* **2015**, *34*, 107–115, doi:10.1016/j.jes.2015.01.029.
88. Bilandžić, N.; Sedak, M.; Čalopek, B.; Džafić, N.; Ostojić, D.M.; Potočnjak, D. Metal Content in Four Shellfish Species from the Istrian Coast of Croatia. *Bull. Environ. Contam. Toxicol.* **2015**, *95*, 611–617, doi:10.1007/s00128-015-1619-0.
89. Norton, G.J.; Deacon, C.M.; Mestrot, A.; Feldmann, J.; Jenkins, P.; Baskaran, C.; Meharg, A.A. Cadmium and Lead in Vegetable and Fruit Produce Selected from Specific Regional Areas of the UK. *Sci. Total Environ.* **2015**, *533*, 520–527, doi:10.1016/j.scitotenv.2015.06.130.
90. Bian, B.; suo Wu, H.; Lv, L.; Fan, Y.; Lu, H. Health Risk Assessment of Metals in Food Crops and Related Soils Amended with Biogas Slurry in Taihu Basin: Perspective from Field Experiment. *Environ. Sci. Pollut. Res.* **2015**, *22*, 14358–14366, doi:10.1007/s11356-015-4853-4.
91. Ke, S.; Cheng, X.-Y.; Zhang, N.; Hu, H.-G.; Yan, Q.; Hou, L.-L.; Sun, X.; Chen, Z.-N. Cadmium Contamination of Rice from Various Polluted Areas of China and Its Potential Risks to Human Health. *Environ. Monit. Assess.* **2015**, *187*, 408,

doi:10.1007/s10661-015-4638-8.
92. Dziubanek, G.; Piekut, A.; Rusin, M.; Baranowska, R.; Hajok, I. Contamination of Food Crops Grown on Soils with Elevated Heavy Metals Content. *Ecotoxicol. Environ. Saf.* **2015**, *118*, 183–189, doi:10.1016/j.ecoenv.2015.04.032.
93. Zhao, K.; Fu, W.; Ye, Z.; Zhang, C. Contamination and Spatial Variation of Heavy Metals in the Soil-Rice System in Nanxun County, Southeastern China. *Int. J. Environ. Res. Public Health* **2015**, *12*, 1577–1594, doi:10.3390/ijerph120201577.
94. Naseri, M.; Vazirzadeh, A.; Kazemi, R.; Zaheri, F. Concentration of Some Heavy Metals in Rice Types Available in Shiraz Market and Human Health Risk Assessment. *Food Chem.* **2015**, *175*, 243–248, doi:10.1016/j.foodchem.2014.11.109.
95. Mok, J.S.; Kwon, J.Y.; Son, K.T.; Choi, W.S.; Shim, K.B.; Lee, T.S.; Kim, J.H. Distribution of Heavy Metals in Muscles and Internal Organs of Korean Cephalopods and Crustaceans: Risk Assessment for Human Health. *J. Food Prot.* **2014**, *77*, 2168–2175, doi:10.4315/0362-028X.JFP-14-317.
96. Rjeibi, M.; Metian, M.; Hajji, T.; Guyot, T.; Ben Chaouacha-Chekir, R.; Bustamante, P. Seasonal Survey of Contaminants (Cd and Hg) and Micronutrients (Cu and Zn) in Edible Tissues of Cephalopods from Tunisia: Assessment of Risk and Nutritional Benefits. *J. Food Sci.* **2015**, *80*, T199–T206, doi:10.1111/1750-3841.12711.
97. Lin, K.; Lu, S.; Wang, J.; Yang, Y. The Arsenic Contamination of Rice in Guangdong Province, the Most Economically Dynamic Provinces of China: Arsenic Speciation and Its Potential Health Risk. *Environ. Geochem. Health* **2015**, *37*, 353–361, doi:10.1007/s10653-014-9652-1.
98. Basu, N.; Tutino, R.; Zhang, Z.; D, C.; Goodrich, J.M.; Somers, E.C.; Rodriguez, L.; Schnaas, L.; Solano, M.; Mercado, A.; et al. Mercury Levels in Pregnant Women, Children, and Seafood from Mexico City. *Env. Res* **2014**, *135*, 63–69, doi:10.1016/j.envres.2014.08.029.Mercury.
99. Zhang, H.; Chen, J.; Zhu, L.; Yang, G.; Li, D. Transfer of Cadmium from Soil to Vegetable in the Pearl River Delta Area, South China. *PLoS One* **2014**, *9*, e108572, doi:10.1371/journal.pone.0108572.
100. Kohrman, H.; Chamberlain, C.P. Heavy Metals in Produce from Urban Farms in the San Francisco Bay Area. *Food Addit. Contam. Part B* **2014**, *7*, 127–134, doi:10.1080/19393210.2013.859740.
101. Adetunji, V.O.; Famakin, I.O.; Chen, J. Lead and Cadmium Levels in Cattle Muscle and Edible Tissues Collected from a Slaughter Slab in Nigeria. *Food Addit. Contam. Part B* **2014**, *7*, 79–83, doi:10.1080/19393210.2013.848942.
102. Castro-González, N.P.; Moreno-Rojas, R.; Calderón Sánchez, F.; Moreno-Ortega, A.; Tamariz-Flores, J.V. Metales Pesados En Leche de Vacas Alimentadas Con Alfalfa Producida En Suelos Irrigados Con Aguas Residuales En Puebla y Tlaxcala, México. *Rev. Mex. Ciencias Pecu.* **2018**, *9*, 466–485, doi:10.22319/rmcp.v9i3.4358.
103. Babra Moyo; Vhahangwele Matodzi; Malebogo A Legodi; Vusumzi E Pakade; Nikita T Tavengwa Determination of Cd, Mn and Ni Accumulated in Fruits, Vegetables and Soil in the Thohoyandou Town Area, South Africa. *Water SA* **2020**, *46*, doi:10.17159/wsa/2020.v46.i2.8244.
104. Mehouel, F.; Bouayad, L.; Hammoudi, A.H.; Ayadi, O.; Regad, F. Evaluation of the Heavy Metals (Mercury, Lead, and Cadmium) Contamination of Sardine (Sardina Pilchardus) and Swordfish (Xiphias Gladius) Fished in Three Algerian Coasts. *Vet. World* **2019**, *12*, 7–11, doi:10.14202/vetworld.2019.7-11.
105. Ametepey, S.T.; Cobbina, S.J.; Akpabey, F.J.; Duwiejuah, A.B.; Abuntori, Z.N. Health Risk Assessment and Heavy Metal Contamination Levels in Vegetables from Tamale Metropolis, Ghana. *Int. J. Food Contam.* **2018**, *5*, 5, doi:10.1186/s40550-018-0067-0.
106. Majlesi, M.; Malekzadeh, J.; Berizi, E.; Toori, M.A. Heavy Metal Content in Farmed Rainbow Trout in Relation to Aquaculture Area and Feed Pellets. *Foods Raw Mater.* **2019**, 329–338, doi:10.21603/2308-4057-2019-2-329-338.
107. Kribi-Boukhris, S.E.; Boughattas, I.; Zitouni, N.; Helaoui, S.; Sappin-Didier, V.; Coriou, C.; Bussiere, S.; Banni, M. Ecotoxicity of Trace Elements to Chicken GALLUS Gallus Domesticus Exposed to a Gradient of Polymetallic-Polluted Sites. *Environ. Pollut.* **2020**, *265*, 114831, doi:10.1016/j.envpol.2020.114831.
108. Jolly, Y.N.; Iqbal, S.; Rahman, M.S.; Kabir, J.; Akter, S.; Ahmad, I. Energy Dispersive X-Ray Fluorescence Detection of Heavy Metals in Bangladesh Cows' Milk. *Heliyon* **2017**, *3*, e00403, doi:10.1016/j.heliyon.2017.e00403.
109. Alvarenga, P.; Simões, I.; Palma, P.; Amaral, O.; Matos, J.X. Field Study on the Accumulation of Trace Elements by Vegetables Produced in the Vicinity of Abandoned Pyrite Mines. *Sci. Total Environ.* **2014**, *470–471*, 1233–1242, doi:10.1016/j.scitotenv.2013.10.087.
110. Ravanbakhsh, M.; Zare Javid, A.; Hadi, M.; Jaafarzadeh Haghighi Fard, N. Heavy Metals Risk Assessment in Fish Species (Johnius Belangerii (C) and Cynoglossus Arel) in Musa Estuary, Persian Gulf. *Environ. Res.* **2020**, *188*, 109560, doi:10.1016/j.envres.2020.109560.
111. Säumel, I.; Kotsyuk, I.; Hölscher, M.; Lenkereit, C.; Weber, F.; Kowarik, I. How Healthy Is Urban Horticulture in High Traffic Areas? Trace Metal Concentrations in Vegetable Crops from Plantings within Inner City Neighbourhoods in Berlin, Germany. *Environ. Pollut.* **2012**, *165*, 124–132, doi:10.1016/j.envpol.2012.02.019.
112. Zheng, S.; Wang, Q.; Yuan, Y.; Sun, W. Human Health Risk Assessment of Heavy Metals in Soil and Food Crops in the Pearl River Delta Urban Agglomeration of China. *Food Chem.* **2020**, *316*, 126213, doi:10.1016/j.foodchem.2020.126213.
113. Bortey-Sam, N.; Nakayama, S.M.M.; Ikenaka, Y.; Akoto, O.; Baidoo, E.; Yohannes, Y.B.; Mizukawa, H.; Ishizuka, M. Human Health Risks from Metals and Metalloid via Consumption of Food Animals near Gold Mines in Tarkwa, Ghana: Estimation of the Daily Intakes and Target Hazard Quotients (THQs). *Ecotoxicol. Environ. Saf.* **2015**, *111*, 160–167, doi:10.1016/j.ecoenv.2014.09.008.
114. Miedico, O.; Pompa, C.; Moscatelli, S.; Chiappinelli, A.; Carosielli, L.; Chiaravalle, A.E. Lead, Cadmium and Mercury in Canned and Unprocessed Tuna: Six-Years Monitoring Survey, Comparison with Previous Studies and Recommended Tolerable Limits. *J. Food Compos. Anal.* **2020**, *94*, 103638, doi:10.1016/j.jfca.2020.103638.
115. Esposito, M.; Canzanella, S.; Lambiase, S.; Scaramuzzo, A.; La Nucara, R.; Bruno, T.; Picazio, G.; Colarusso, G.; Brunetti, R.; Gallo, P. Organic Pollutants (PCBs, PCDD/Fs, PAHs) and Toxic Metals in Farmed Mussels from the Gulf of Naples (Italy): Monitoring and Human Exposure. *Reg. Stud. Mar. Sci.* **2020**, *40*, 101497, doi:10.1016/j.rsma.2020.101497.
116. Adeogun, A.O.; Ibor, O.R.; Omiwole, R.; Chukwuka, A. V.; Adewale, A.H.; Kumuyi, O.; Arukwe, A. Sex-Differences in Physiological and Oxidative Stress Responses and Heavy Metals Burden in the Black Jaw Tilapia, Sarotherodon Melanotheron from a Tropical Freshwater Dam (Nigeria). *Comp. Biochem. Physiol. Part C Toxicol. Pharmacol.* **2020**, *229*, 108676, doi:10.1016/j.cbpc.2019.108676.
117. He, M.; Shen, H.; Li, Z.; Wang, L.; Wang, F.; Zhao, K.; Liu, X.; Wendroth, O.; Xu, J. Ten-Year Regional Monitoring of Soil-Rice Grain Contamination by Heavy Metals with Implications for Target Remediation and Food Safety. *Environ. Pollut.* **2019**, *244*, 431–439, doi:10.1016/j.envpol.2018.10.070.
118. Mok, J.S.; Kwon, J.Y.; Son, K.T.; Choi, W.S.; Kang, S.R.; Ha, N.Y.; Jo, M.R.; Kim, J.H. Contents and Risk Assessment of Heavy Metals in Marine Invertebrates from Korean Coastal Fish Markets. *J. Food Prot.* **2014**, *77*, 1022–1030, doi:10.4315/0362-028X.JFP-13-485.
119. Najarnezhad, V.; Akbarabadi, M. Heavy Metals in Raw Cow and Ewe Milk from North-East Iran. *Food Addit. Contam. Part B* **2013**, *6*, 158–162, doi:10.1080/19393210.2013.777799.
120. Jokanović, M.R.; Tomović, V.M.; Šojić, B.V.; Škaljac, S.B.; Tasić, T.A.; Ikonić, P.M.; Kevrešan, Ž.S. Cadmium in Meat and Edible Offal of Free-Range Reared Swallow-Belly Mangulica Pigs from Vojvodina (Northern Serbia). *Food Addit. Contam. Part B* **2013**, *6*, 98–102, doi:10.1080/19393210.2012.729227.
121. Luis, G.; Rubio, C.; González-Weller, D.; Gutiérrez, A.J.; Revert, C.; Hardisson, A. Evaluation of Content and Estimation of Daily Intake of Cadmium and Lead in Several Varieties of Potatoes (Solanum Tuberosum L.) Cultivated in the Canary Islands (Spain). *J. Food Prot.* **2014**, *77*, 659–664, doi:10.4315/0362-028X.JFP-13-337.
122. von Hoffen, L.P.; Säumel, I. Orchards for Edible Cities: Cadmium and Lead Content in Nuts, Berries, Pome and Stone Fruits Harvested within the Inner City Neighbourhoods in Berlin, Germany. *Ecotoxicol. Environ. Saf.* **2014**, *101*, 233–239, doi:10.1016/j.ecoenv.2013.11.023.
123. Rahman, M.A.; Rahman, M.M.; Reichman, S.M.; Lim, R.P.; Naidu, R. Heavy Metals in Australian Grown and Imported Rice and Vegetables on Sale in Australia: Health Hazard. *Ecotoxicol. Environ. Saf.* **2014**, *100*, 53–60, doi:10.1016/j.ecoenv.2013.11.024.
124. Chang, C.Y.; Yu, H.Y.; Chen, J.J.; Li, F.B.; Zhang, H.H.; Liu, C.P. Accumulation of Heavy Metals in Leaf Vegetables from Agricultural Soils and Associated Potential Health Risks in the Pearl River Delta, South China. *Environ. Monit. Assess.* **2014**, *186*, 1547–1560, doi:10.1007/s10661-013-3472-0.
125. Zhou, H.; Zeng, M.; Zhou, X.; Liao, B.-H.; Liu, J.; Lei, M.; Zhong, Q.-Y.; Zeng, H. Assessment of Heavy Metal Contamination and Bioaccumulation in Soybean Plants from Mining and Smelting Areas of Southern Hunan Province, China. *Environ. Toxicol. Chem.* **2013**, *32*, 2719–2727, doi:10.1002/etc.2389.
126. Heidari, B.; Riyahi Bakhtiari, A.; Shirneshan, G. Concentrations of Cd, Cu, Pb and Zn in Soft Tissue of Oyster (Saccostrea Cucullata) Collected from the Lengeh Port Coast, Persian Gulf, Iran: A Comparison with the Permissible Limits for Public Health. *Food Chem.* **2013**, *141*, 3014–3019, doi:10.1016/j.foodchem.2013.06.002.
127. Xia, C.; Wu, X.; Lam, J.C.W.; Xie, Z.; Lam, P.K.S. Methylmercury and Trace Elements in the Marine Fish from Coasts of East China. *J. Environ. Sci. Heal. Part A* **2013**, *48*, 1491–1501, doi:10.1080/10934529.2013.796820.
128. Olmedo, P.; Pla, A.; Hernández, A.F.; Barbier, F.; Ayouni, L.; Gil, F. Determination of Toxic Elements (Mercury, Cadmium, Lead, Tin and Arsenic) in Fish and Shellfish Samples. Risk Assessment for the Consumers. *Environ. Int.* **2013**, *59*, 63–72, doi:10.1016/j.envint.2013.05.005.
129. Al-Mughairi, S.; Yesudhason, P.; Al-Busaidi, M.; Al-Waili, A.; Al-Rahbi, W.A.K.; Al-Mazrooei, N.; Al-Habsi, S.H. Concentration and Exposure Assessment of Mercury in Commercial Fish and Other Seafood Marketed in Oman. *J. Food Sci.* **2013**, *78*, T1082–T1090, doi:10.1111/1750-3841.12150.
130. Xu, D.; Zhou, P.; Zhan, J.; Gao, Y.; Dou, C.; Sun, Q. Assessment of Trace Metal Bioavailability in Garden Soils and Health Risks via Consumption of Vegetables in the Vicinity of Tongling Mining Area, China. *Ecotoxicol. Environ. Saf.* **2013**, *90*, 103–111, doi:10.1016/j.ecoenv.2012.12.018.
131. Batista, B.L.; Grotto, D.; Carneiro, M.F.H.; Barbosa, F. Evaluation of the Concentration of Nonessential and Essential Elements in Chicken, Pork, and Beef Samples Produced in Brazil. *J. Toxicol. Environ. Heal. Part A* **2012**, *75*, 1269–1279, doi:10.1080/15287394.2012.709439.
132. Salazar, M.J.; Rodriguez, J.H.; Nieto, G.L.; Pignata, M.L. Effects of Heavy Metal Concentrations (Cd, Zn and Pb) in Agricultural Soils near Different Emission Sources on Quality, Accumulation and Food Safety in Soybean [Glycine Max (L.) Merrill]. *J. Hazard. Mater.* **2012**, *233–234*, 244–253, doi:10.1016/j.jhazmat.2012.07.026.
133. Li, Q.; Chen, Y.; Fu, H.; Cui, Z.; Shi, L.; Wang, L.; Liu, Z. Health Risk of Heavy Metals in Food Crops Grown on Reclaimed Tidal Flat Soil in the Pearl River Estuary, China. *J. Hazard. Mater.* **2012**, *227–228*, 148–154, doi:10.1016/j.jhazmat.2012.05.023.
134. Cheraghi, M.; Lorestani, B.; Merrikhpour, H.; Rouniasi, N. Heavy Metal Risk Assessment for Potatoes Grown in Overused Phosphate-Fertilized Soils. *Environ. Monit. Assess.* **2013**, *185*, 1825–1831, doi:10.1007/s10661-012-2670-5.
135. Shao, D.D.; Wu, S.C.; Liang, P.; Kang, Y.; Fu, W.J.; Zhao, K.L.; Cao, Z.H.; Wong, M.H. A Human Health Risk Assessment of Mercury Species in Soil and Food around Compact Fluorescent Lamp Factories in Zhejiang Province, PR China. *J.*

Hazard. Mater. **2012**, *221–222*, 28–34, doi:10.1016/j.jhazmat.2012.03.061.
136. Lourenço, H.M.; Afonso, C.; Anacleto, P.; Martins, M.F.; Nunes, M.L.; Lino, A.R. Elemental Composition of Four Farmed Fish Produced in Portugal. *Int. J. Food Sci. Nutr.* **2012**, *63*, 853–859, doi:10.3109/09637486.2012.681632.
137. Al-Rmalli, S.W.; Jenkins, R.O.; Haris, P.I. Dietary Intake of Cadmium from Bangladeshi Foods. *J. Food Sci.* **2012**, *77*, T26–T33, doi:10.1111/j.1750-3841.2011.02467.x.
138. Al-Busaidi, M.; Yesudhason, P.; Al-Mughairi, S.; Al-Rahbi, W.A.K.; Al-Harthy, K.S.; Al-Mazrooei, N.A.; Al-Habsi, S.H. Toxic Metals in Commercial Marine Fish in Oman with Reference to National and International Standards. *Chemosphere* **2011**, *85*, 67–73, doi:10.1016/j.chemosphere.2011.05.057.
139. Yang, Q.; Xu, Y.; Liu, S.; He, J.; Long, F. Concentration and Potential Health Risk of Heavy Metals in Market Vegetables in Chongqing, China. *Ecotoxicol. Environ. Saf.* **2011**, *74*, 1664–1669, doi:10.1016/j.ecoenv.2011.05.006.
140. Lei, L.; Liang, D.; Yu, D.; Chen, Y.; Song, W.; Li, J. Human Health Risk Assessment of Heavy Metals in the Irrigated Area of Jinghui, Shaanxi, China, in Terms of Wheat Flour Consumption. *Environ. Monit. Assess.* **2015**, *187*, 647, doi:10.1007/s10661-015-4884-9.
141. Burioli, E.A.V.; Squadrone, S.; Stella, C.; Foglini, C.; Abete, M.C.; Prearo, M. Trace Element Occurrence in the Pacific Oyster Crassostrea Gigas from Coastal Marine Ecosystems in Italy. *Chemosphere* **2017**, *187*, 248–260, doi:10.1016/j.chemosphere.2017.08.102.
142. Chijioke, N.O.; Uddin Khandaker, M.; Tikpangi, K.M.; Bradley, D.A. Metal Uptake in Chicken Giblets and Human Health Implications. *J. Food Compos. Anal.* **2020**, *85*, 103332, doi:10.1016/j.jfca.2019.103332.
143. Damerau, A.; Venäläinen, E.R.; Peltonen, K. Heavy Metals in Meat of Finnish City Rabbits. *Food Addit. Contam. Part B* **2012**, *5*, 246–250, doi:10.1080/19393210.2012.702131.
144. Hassan, A.A.; Brustad, M.; Sandanger, T.M. Concentrations and Geographical Variations of Selected Toxic Elements in Meat from Semi-Domesticated Reindeer (Rangifer Tarandus Tarandus L.) in Mid- and Northern Norway: Evaluation of Risk Assessment. *Int. J. Environ. Res. Public Health* **2012**, *9*, 1699–1714, doi:10.3390/ijerph9051699.
145. Zupka, S.; Vollmannová, A.; Harangozo, Ľ.; Slávik, M.; Medvecký, M. Risk of Contamination of Wild Berries from Upper Orava Region by Cadmium. *Potravin. Slovak J. Food Sci.* **2016**, *10*, 126–131, doi:10.5219/546.
146. Filippini, T.; Tancredi, S.; Malagoli, C.; Cilloni, S.; Malavolti, M.; Violi, F.; Vescovi, L.; Bargellini, A.; Vinceti, M. Aluminum and Tin: Food Contamination and Dietary Intake in an Italian Population. *J. Trace Elem. Med. Biol.* **2019**, *52*, 293–301, doi:10.1016/j.jtemb.2019.01.012.
147. Akele, M.L.; Abebe, D.Z.; Alemu, A.K.; Assefa, A.G.; Madhusudhan, A.; de Oliveira, R.R. Analysis of Trace Metal Concentrations in Raw Cow's Milk from Three Dairy Farms in North Gondar, Ethiopia: Chemometric Approach. *Environ. Monit. Assess.* **2017**, *189*, 499, doi:10.1007/s10661-017-6203-0.
148. Xu, X.; Han, J.; Abeysinghe, K.S.; Atapattu, A.J.; De Silva, P.M.C.S.; Xu, Z.; Long, S.; Qiu, G. Dietary Exposure Assessment of Total Mercury and Methylmercury in Commercial Rice in Sri Lanka. *Chemosphere* **2020**, *239*, 124749, doi:10.1016/j.chemosphere.2019.124749.
149. Brizio, P.; Benedetto, A.; Squadrone, S.; Curcio, A.; Pellegrino, M.; Ferrero, M.; Abete, M.C. Heavy Metals and Essential Elements in Italian Cereals. *Food Addit. Contam. Part B* **2016**, *9*, 261–267, doi:10.1080/19393210.2016.1209572.
150. Zhao, H.; Yan, H.; Zhang, L.; Sun, G.; Li, P.; Feng, X. Mercury Contents in Rice and Potential Health Risks across China. *Environ. Int.* **2019**, *126*, 406–412, doi:10.1016/j.envint.2019.02.055.
151. Potí, P.; Pajor, F.; Bodnár, Á.; Bárdos, L. Accumulation of Some Heavy Metals (Pb, Cd and Cr) in Milk of Grazing Sheep in North-East Hungary. *J. Microbiol. Biotechnol. Food Sci.* **2012**, *2*, 389–394.
152. Ihedioha, J.N.; Ekere, N.R.; Okoye, C.O.B. Cadmium in Locally Grown Rice (*Oryza Sativa*) in Nigeria. *Food Addit. Contam. Part B* **2013**, *6*, 275–278, doi:10.1080/19393210.2013.821174.
