# Supplementary material for: Heavy Metals in Unprocessed or Minimally Processed Foods Consumed by Humans Worldwide: A Scoping Review
Source: Int J Environ Res Public Health. 2022 Jul 16;19(14):8651. doi: 10.3390/ijerph19148651 (PMC9319294; doi:10.3390/ijerph19148651)
Supplement: Supplementary file 1 [file ijerph-19-08651-s001.zip › Table S1_Classification of individual Foods.pdf]

Table S1. Classification of our individual Food items according to the Global Individual Information Food Consumption Data Tool (FAO/WHO GIFT)

Food group code	Food group	Subgroup- short name	Food group description*
1	Cereals	Rice	Rice types (husked, unpolish and polished, white, red, brown, black, basmati) and rice products (semolina, flour, bran, popped rice, rolled grains and porridge)
		Maize	Maize types and milled grain
		Weat	Wheat, including secondary commodities and derived products such as groats, semolina, flour, bran, wheat grain germ, rolled and popped grains and porridge
		Sorghum	Sorghum, including secondary commodities and derived products such as flour
		Millet	Millet and products as groats, flour and rolled grains
		Other and mixed cereals	Grains other than rice, maize, wheat, sorghum and millet, such as barley, buckwheat, oat, and rye
2	Roots, tubers	Potato	Potato and sweet potato types
		Cassava	Cassava and similar roots
		Taro	Taro (Colocasia esculenta (L.) Schott)
		Yam	Yam (Dioscorea L. spp.)
		Other starchy roots and tubers	Starchy roots and tubers other than potato and sweet potato, cassava and similar roots, taro and yam
3	Pulses, seeds and nuts	Pulses	Dry beans (excluding soybeans), peas, lentils, lupins and other or unspecified pulses and their sprouts
		Soybeans	Dry soybeans and their sprouts
		Nuts and seeds	Nuts and seeds such as tree nuts (almonds, cashew nuts, chestnuts, coconuts, walnuts and similar)
4	Milk	Milk (specify animal origin)	Milk types: cow, sheep and goat
5	Eggs	Eggs (specify animal origin)	Egg origin types: chicken, turtle, fish, and duck
6	Fish and shellfish	Freshwater fish	Meat from freshwater fish
		Diadromous fish	Meat from diadromous fish
		Marine fish	Meat from marine fish
		Offal	Offal from all types of fish and shellfish
		Crustacean	Prawn, shrimp, crab and, lobster

		Mollusks	Clams, cockles and mussels
		Cephalopods	Cuttlefishes, octopuses and squids without viscera, squid
7	Meat	Red meat / mammals, reptiles and amphibians	Mammal meat (excluding offal); bovine, pork, equine, goat, sheep, camelid, deer, rabbit, kangaroo, meat from rodents, meat from reptiles and amphibians such as snails, crocodiles, frogs, snakes, turtles and lizards
		Poultry	Bird meat (excluding offal); chicken, turkey, duck, goose, pigeon, ratites, quail and pheasant
		Offal red meat	Organ meat such as from the liver, kidney, heart, lungs, intestines and blood of red meat subgroup
		Offal poultry	Organ meat such as from the liver, kidney, heart, lungs, intestines and blood of poultry subgroup
8	Vegetables	Leafy vegetables	Lettuce, rocket salad, mustard leaves, bay leaves, spinach and similar, chard and similar, whitlow grass, cabbage and Brussels sprouts, kale, other leafy brassica and ferns, and seaweeds such as algae
		Stalk and steem vegetables	Celery and asparagus: remove adhering soil.
		Brassica vegetables	Head cabbages, kohlrabi, Cauliflower and broccoli: flower heads (immature inflorescence only). Brussels sprouts: “buttons” only, cabbage,
		Bulb vegetables	fresh garlic, onion and similar, leek, Chinese chive, scallion
		Fruiting vegetables	Whole commodity after removal of stems. Sweet corn and fresh corn: kernels plus cob without husk. Tomato, pumpkin, eggplant,
		Legume vegetable	The succulent forms may be consumed as whole pods or as the shelled product: pods, peas, green beans, broadbean, dolichos lablab, edible pods, green cowpea
9	Fruits	Tropical fruits	avocado, banana, carambola, guavas, mangoes, papaya, pineaple,
		Stone fruits	apricots, cherries, dates, nectarines, olive, peaches, plumps
		Pome fruits	apples, pears, quinces, okra
		Citrus fruits	lemon, grapefruits, limes, mandarins, oranges, tangerines
		watermelons fruits	Citrullus lanatus, Cucumis melo,
		Soft fruits	blueberries, blackberries, strawberries, raspberries, grapes,

Adapted from General Standards for Contaminants and Toxins in Food and Feed. Amended in 2019, and from Ultra-processed foods, diet quality and health using NOVA classification system and AGROVOC Multilingual Thesaurus FAO.
*Foods reported in the articles included in this Scoping Review.
